# Powdered Beverage from Native Plants from Argentina (*Zuccagnia punctata* and *Solanum betaceum*) Obtained by Spray-Drying: A Promising Source of Antioxidant Compounds

**DOI:** 10.3390/plants12081646

**Published:** 2023-04-14

**Authors:** Florencia María Correa Uriburu, Iris Catiana Zampini, Luis Maria Maldonado, Milagros Gómez Mattson, Daniela Salvatori, María Inés Isla

**Affiliations:** 1 Instituto de Bioprospección y Fisiología Vegetal (INBIOFIV), CONICET—Universidad Nacional de Tucumán (UNT), San Miguel de Tucumán T4000CBG, Argentina; florcorreau@gmail.com (F.M.C.U.); zampini@csnat.unt.edu.ar (I.C.Z.); 2 Instituto Nacional de Tecnología Agropecuaria, Estación Experimental Agropecuaria, Famaillá (INTA), Ruta Provincial 301-km 32, Famaillá 4132, Tucumán, Argentina; maldonado.luismaria@inta.gob.ar; 3 Facultad de Ciencias Naturales e Instituto Miguel Lillo, Universidad Nacional de Tucumán (UNT), San Miguel de Tucumán T4000JFE, Argentina; 4 Instituto de Investigación y Desarrollo en Ingeniería de Procesos, Biotecnología y Energías Alternativas (PROBIEN), Neuquén 8300, Argentina; milagros.gomez@probien.gob.ar (M.G.M.); daniela.salvatori@probien.gob.ar (D.S.)

**Keywords:** *Z. punctata*, *S. betaceum*, functional beverages, powder, phenolic compounds, flavonoids, antioxidant capacity

## Abstract

In previous studies, the Argentinean native plants called *Zuccagnia punctata* (jarilla, pus pus, lata) and *Solanum betaceum* (chilto, tree tomato) were reported as new natural sources of antioxidant compounds, mainly chalcones, anthocyanins and rosmarinic acid derivates. The present study deals with the production of antioxidant beverages of *Z. punctata* (Zp) extract and chilto juice with honey as sweetener. A Zp extract and red chilto juice were obtained according to Food Code and characterized. The beverages were formulated by using maltodextrin (MD) with two dextrose equivalents (DE), 10 and 15, and then spray-dried at an inlet air temperature of 130 °C. The physicochemical, microscopical, phytochemical and functional characteristics of the powders were surveyed. The experiments carried out showed good physical properties for both formulations showing high water solubility with adequate features for handling, transport and storage. The chromatic parameters of both powdered beverages indicate orange–pink tones regardless of the wall material used. The total polyphenol and flavonoid content in the beverages were kept after spray-drying (92 and 100%, respectively). The anthocyanins were less stable under drying conditions (yield 58%). Both powdered beverages showed high scavenger capacity on ABTS^•+^, HO^•^ and H_2_O_2_ (SC_50_ between 3.29 to 41.05 µg GAE/mL) and were able to inhibit xanthine oxidase (XOD) activity (CI_50_ between 91.35 and 114.43 µg GAE/mL). The beverages were neither toxic nor mutagenic in the concentration range with biological activity. The results obtained in the present work scientifically support the use of the powdered beverages of Argentinean native plants as antioxidant.

## 1. Introduction

Polyphenols are plant metabolites that are commonly found in food and medicinal plants. These compounds are responsible for several functional properties closely related with the prevention of certain oxidative processes, which are, in turn, responsible for inflammatory pathologies, cancer, neurodegenerative and cardiovascular diseases [1,2]. They also improve the intestinal barrier integrity down-regulating various inflammatory molecules [3] and modulate the intestinal microbiota [4]. Apart from the aforementioned functional traits, these metabolites play a central role in several sensory characteristics such as colour, flavour, bitterness and astringency in beverages, such as beer, cider, wine and tea or plant infusions, as well as in foods [5]. In addition, several polyphenolic pigments such as anthocyanins, flavanols and flavones are involved in food flavour [6,7]. Argentine native fruits such as *Solanum betaceum* Cav and medicinal plants from Argentina, such as *Zuccagnia punctata* Cav contain several polyphenolic metabolites with multiple functional properties, such as antioxidant, anti-inflammatory, antitumoral, antimicrobial, among others.

**Figure 1:**
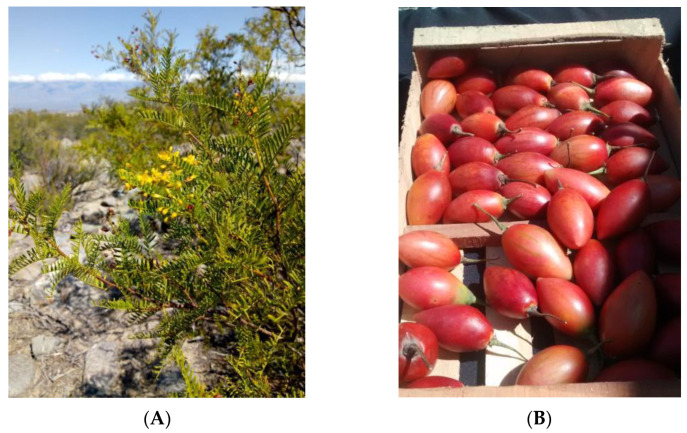
(**A**) Aerial parts of *Z. punctata* Cav. (**B**) *S. betaceum* fruits, Photo by F. M. Correa Uriburu.

*Zuccagnia punctata* Cav (Fabaceae) is an Argentine endemic medicinal plant. It is a glutinous and aromatic shrub [8] (Figure 1A). The infusion and decoction in water, as well as maceration in ethanol of aerial parts of *Z. punctata*, with and without flowers or fruits, have been used extensively as a traditional medicine in Argentina as foot antiseptic and rubefacient, and against bacterial and fungal infections, asthma, arthritis, rheumatism, inflammations, and tumors [9]. A wide range of biological activities, such as antibacterial, antifungal, nematicidal, cytoprotective, anti-inflammatory, antioxidant, antitumoral, hypoglucemic and antihypertensive were previously described, to leaves, stems and flowers extracts [8,9,10,11,12,13,14,15,16,17,18,19,20,21,22,23,24,25,26,27,28,29,30,31,32]. It is also worth mentioning that it has a significant effect on the prevention of cardiovascular diseases related with hypercholesterolemia and endothelial dysfunction [14,16]. No genotoxic effect of *Z. punctata* extracts has been demonstrated [27]. Oral administration of *Z. punctata* extract and some of their isolated compounds were not toxic in rabbits and mice models [16,24]. Apart from several phenolic acids and flavonoids, the main bioactive phytochemicals in plant aerial organs were identified as 2′,4′-dihydroxy-3′-methoxy chalcone (DHMC) and 2′,4′-dihydroxychalcone (DHC), which were proposed as bioactive chemical markers [8,9,10,11,12,13,14,15,16,17,18,19,20,21,22,23,24,25,26,27,28,29,30,31,32]. Therefore, extracts of aerial parts of *Z. punctata* could be used to make tea or infusions, macerations and other phytotherapeutic and food preparations such as functional beverages.

*Solanum betaceum* Cav or chilto, or tomato tree is a food species native to tropical or subtropical regions from Colombia to Argentina. In the late 19th century, the fruit was globally introduced in Australia and New Zealand, South-East Asia and Europe [33]. New Zealand and Portugal are today the main producers and exporters. Countries such as the United States, Japan, Hong Kong, Singapore, Australia and the Pacific Islands are the prime international markets for *S. betaceum* fruits [34]. The berry-like fruits are fleshy, juicy and bittersweet. The pulp and skin present different colours: yellow, orange and red (Figure 1B). The fruits are used in a wide range of foods, namely, salads, jams, juices and liquors, as well as in medicinal preparations aimed to alleviate the symptoms of anemia, liver and respiratory diseases, obesity, cholesterol and inflammation diseases [35,36]. The fruits of *S. betaceum* have been extensively studied in terms of their chemical composition, as well as their biological and functional activity. Chemical studies revealed that chilto fruits are rich in fiber, potassium, ascorbic acid and carotenoids, while components such as carbohydrates and lipids are present in smaller amounts [34,36,37,38,39,40,41,42]. Several beneficial health activities have been reported as anti-oxidative, anti-proliferative, anti-nociceptive, anti-inflammatory, anti-obesity, and antimicrobial [34,36,37,38,39,43,44]. Many of these reported properties are due to the presence of phenolic compounds, such as derivatives of rosmarinic acid, 3-O-caffeoylquinic acid, or chlorogenic acid such as hydroxycinnamic acids [36,37,38,43]. In the case of the red variety, the predominant polyphenolic compound in the pulp is anthocyanin, a hydrosoluble pigment, while hexanoic acid methyl ester is the main volatile compound [44,45]. Biodegradable food packaging by using polyphenols and polysaccharides from *S. betaceum* was developed [46]. Recently, films containing anthocyanin-enriched extracts obtained from *S. betaceum* red fruits were obtained by casting [47]. Several food products such as frozen functional pulps, energy and effervescent drinks and ice creams have been developed from *S. betaceum* pulp [38,47,48,49,50]. The demonstrated properties, mainly the antioxidant activity of both plant species, make them very promising for the formulation of functional beverages.

Some bioactive phenolic compounds are highly unstable during processing and storage. Hence, a technology to protect them is necessary. Spray-drying is the most common process used for microencapsulation of active ingredients for the protection from environmental conditions. Numerous materials, which include proteins, gums and modified starches, have been used as encapsulating agents [51,52]. Maltodextrin (MD) is a partially hydrolyzed starch that has been frequently used as a reference wall material for the encapsulation of antioxidants because it is an abundant and low-cost encapsulating agent that has high water solubility and is able to protect encapsulated ingredients from oxidation [51,52,53,54]. Based on the aforementioned analysis, the present research focuses on the obtention and characterization of an antioxidant powdered beverage of *Z. punctata* and *S. betaceum* by applying a spray-drying process and using different types of MD as wall material.

## 2. Results and Discussion

According to Bursal et al. [55], the antioxidant potential consists in the ability of a compound to interact with free radicals or non-free radical species and delay or prevent the oxidative process. Thus, a functional beverage rich in antioxidant compounds can be helpful to combat radical species present in the human body in a preventive way. Previous reports showed a high antioxidant potential of infusions made with aerial parts of *Z. punctata* [9,10,15]. Zp infusions and tinctures can be active as antioxidant (ABTS radical cation and hydroxyl radical scavenging capacity) [9,10,14,15,16,19,26]. The antioxidant potential of Zp infusions was similar to that exhibited by black tea (*C. sinensis*), a well-known beverage [15]. Several authors have already demonstrated that some major phytochemicals present in *Z. punctata* extracts, i.e., 7-hydroxyflavanone, 3′,7′-dihydroxyflavone, DHC and DHMC are responsible for free radical scavenging capacities and singlet oxygen quenching [10,14,15,16,19,26,56].

On the other hand, the red fruits of another plant native to South America, chilto, have shown a very important antioxidant capacity such as scavenging of different free radicals, i.e., hydroxyl, superoxide, or hydrogen peroxide [36,37,38,43,46,47,49]. Rosmarinic acid and its derivate, as well as caffeoylquinic acid, were held responsible for the antioxidant activity, both in vitro, before and after simulated gastroduodenal digestion and in vivo experiments [34,36,37,38,57,58,59].

In addition to their antioxidant capacity, both native plant species *(Z. punctata* and *S. betaceum*) have a proven effect on pathologies linked to oxidative stress, i.e., metabolic syndrome. The extracts and powders obtained from them might help lower cholesterol and blood glucose [13,14,16,19,36,37,38,60]. Furthermore, infusion of *Zuccagnia punctata* reduced intestinal transit in rats and mice and offered protection against ethanol-induced ulceration in rats [31]. Both chalcones isolated from *Zuccagnia punctata* showed significant preventive effects on ethanol-induced gastroduodenal injury [61].

These plants represent a source of novel compounds with promising antioxidant activity and other linked properties for exploration of functional non-conventional beverage formulations and provides opportunities for new food markets, as well as meeting consumers’ demands.

Because of all this, a Zp extract and red chilto juice were obtained and characterized in the present study to develop a non-conventional antioxidant non-alcoholic beverage.

### 2.1. Z. punctata Extract Preparation and Chemical Characterization

**Figure 2:**
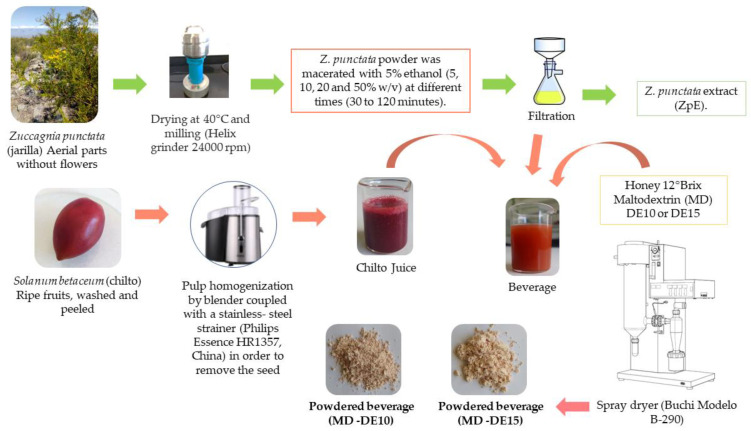
Diagram describing obtention procedure of both *Zuccagnia punctata* extract and chilto juice beverages.

**Figure 3:**
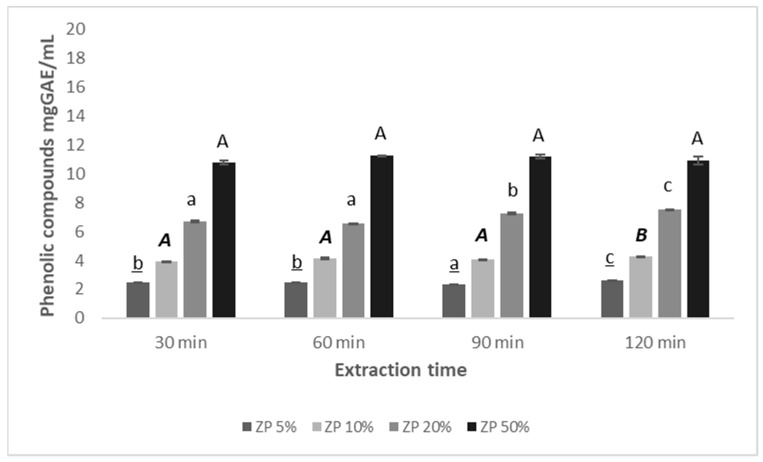
Efficiency in the extraction of phenolic compounds of *Z. punctata* aerial parts by using 5, 10, 20 and 50% plant material in 5% ethanol with different contact time (30, 60, 90 and 120 min). Different letters between bars for the same value of dry weight of *Z. punctata* indicate significant differences between phenolic compounds content at different time, according to Tukey’s test (*p* ≤ 0.05). Capital letter (A,B), lowercase letter (a,b,c), capital and italic letter (***A***,***B***), lowercase and underlined (a,b,c) are used to Zp 50% Zp 20%, Zp 10% and Zp 5%, respectively.

Both the method and solvents, as well as the conditions of extraction of plant biomass, play a key role in the chemical composition of extracts, and consequently in their biological activity. Hence, Zp extracts were obtained from aerial parts of *Z. punctata* by using different quantities of dry herbal material (5, 10, 20 and 50% *w*/*v*) in ethanol 5% with different contact time between plant material and solvent (30 to 120 min) according to the flux diagram in Figure 2. Ethanol 5% was used according to recommendations of the Argentine Food Code, Chapter XII, Article 996, for non-carbonated non-alcoholic beverages [62]. A positive relationship between the content of phenolic or flavonoid compounds extracted with 5% ethanol and the amount of plant matrix used was observed each time. However, no significant difference in the content of these metabolites using different matrix–solvent ratios with equal contact times was found (Figure 3).

Hence, plant extracts containing 20% (*w*/*v*) herbal material extracted in 5% ethanol for 30 min were selected to be used in beverage formulation. The Zp extract 20% (*w*/*v*) showed a 10 times higher level of total and reducing sugars than Zp tea previously characterized by Carabajal et al., 2019 [10], Table 1. The results indicate that in our experimental conditions, ethanol 5% was a better solvent than hot water (infusion) for the reducing sugars. Probably, the ethanol extracts, in addition to the free sugars, some glycosides. Sugars are important regarding flavor as they cause a strong impact on the organoleptic quality of the beverage [63]. The total phenolic compounds and flavonoid content were 6.68 mg GAE/mL (33.4 mg GAE/g) and 12.10 mg QE/mL (60.5 mg QE/g), respectively, similar to the values previously reported for *Z*. *punctata* infusion or tea [10]. Furthermore, condensed and hydrolysable tannins were also detected in the Zp extract. These compounds are responsible for astringent flavors. Anthocyanins, pigments present in *Z. punctata* fruits, were not detected in the Zp extract obtained from aerial parts without flowers and fruits. The phenolic profile of Zp extract 20% (*w*/*v*) in 5% ethanol was similar to that of Zp infusion [10] with two main phenolic compounds, DHC and DHMC. The content of DHC (11.28 μg/mL) was similar to the reported content for infusion (10.9 μg/mL) [10] while DHMC content in Zp extract 20% in ethanol 5% was higher than the values found in Zp infusion (23.22 and 10.1 μg/mL, respectively), Table 1. 

### 2.2. Chilto Juice Characterization

The chemical composition of red chilto juice was determined (Table 1). The sugar content in it was thirteen times higher than in *Z punctata* extract. The content of anthocyanin, phenolic compounds and flavonoids was similar to those previously reported for red chilto pulp [38]. Hydrolysable and condensed tannin was also detected, but the level was lower than in Zp extract (17.8 and 3 times, respectively). The phenolic profile of chilto juice was similar to that of pulp. Two main compounds, rosmarinic acid and caffeoylquinic acid, were identified by HPLC-DAD and the content of them was included in Table 1, 73.63 and 33.67 µg/mL, respectively. Other authors had reported that the total content of rosmarinic acid and caffeoylquinic acid in red chilto fruit powder was 0.291 g/100 g powder and 0.354 g/100 g powder, respectively, similar values to those found in chilto juice [37]. According to the literature, CQA and RA which are present in red chilto have compounds with high antioxidant activity and capacity for the inhibition of digestive enzymes [36,37,38,57,58,59].

### 2.3. Honey Characterization

Honey is a bee product that has been used as a functional food for years for its beneficial effects for human health. Although the great majority of the dry weight of honey (95–98%) consists of carbohydrates, 2–5% is made up of various secondary metabolites and minerals [64,65]. The honey was included in several beverages in different concentrations by its sugar content and bioactive components, principally polyphenolic compounds [66,67]. So, in this work, commercial lemon honey was diluted at 12° Brix and chemically characterized to incorporate it into the antioxidant beverage of native plants of Argentina. The total sugars and reducing sugars level were higher than red chilto juice and *Z. punctata* extract (Table 1), but the phenolic compounds content was low. Flavonoids, tannins and anthocyanins were not detected in our experimental conditions.

### 2.4. Beverage Formulation and Chemical Characterization

A formulation containing *Z. punctata* extract (9%) and red chilto juice (26%), used as natural antioxidant sources, was made for the first time. Honey was included in several beverages by its content in sugar and polyphenol [66,67]. In the present work, lemon honey (12 °Brix of soluble solids) was included as a sweetener (Table 1). The honey concentration was selected according to the recommendations of the Argentine Food Code for non-alcoholic beverages [62]. To address the formulation of an antioxidant beverage, spray-drying was used as a technique for microencapsulation of bioactives from chilto juice and Zp extract, since it is a simple, fast and cost-effective process of droplet-to-particle transition. Due to its ease of transport, storage and application in foods, spray-dried beverage powder is highly convenient; it is also particularly stable in terms of microbiological and chemical degradation due to its low moisture content and low water activity [53,54], not to mention its low hygroscopicity, short dissolution time, as well as high bioactive retention [68]. Powder properties, such as bulk density and bioactive retention, are closely related to the microstructure characteristics of the microcapsules produced during drying [68]. Water-based gel formulations, such as MD are used as coating for polar matrices such as polyphenols. MD is formed by partial hydrolysis, resulting in different values of equivalent dextrose (DE); it presents high solubility in water, low viscosity, low sugar content and produces colorless solutions.

For this, the addition of 15% MD of different DE (10 and 15) was performed to produce microencapsulation by spray-drying (Figure 2). Two powdered beverages were obtained and characterized chemically (Table 2). Fructose, glucose and sucrose are the main soluble sugars (Table 2). Sucrose concentration was higher than that of reductor sugars (glucose and fructose). Each one has a different level of sweetness. The powder with MD-DE 15 retained higher sugar content than powder with MD-DE 10. The protein level was low in both powders. Citric acid and malic acid, two non-volatile organic acids, were detected in the beverage. The level of citric acid was higher in powder with MD-DE 10 than in that with MD-DE 15. Both organic acids were previously detected in the ripe red chilto pulp [37]. The ratio between them (citric acid/malic acid content) was similar in pulp and beverage powders. Malic and citric acids, mild tasting substances used as flavoring and preservative agents in foods, are antioxidant and show antimicrobial capacity [69].

**Figure 4:**
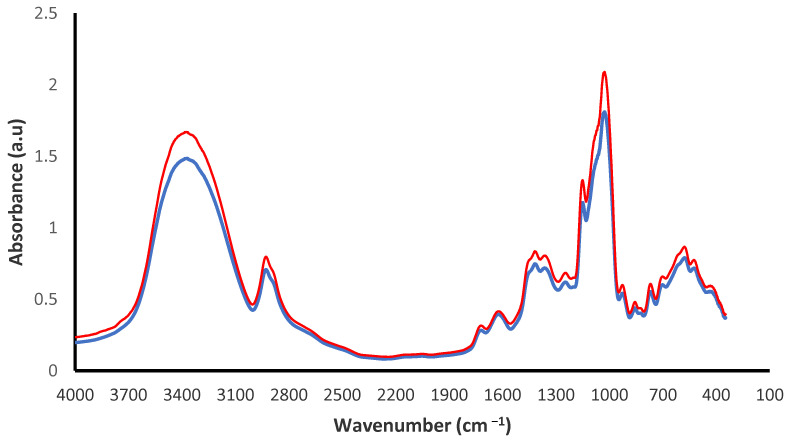
FTIR spectra profiles for beverage powders with MD-DE 10 (red lines) and MD-DE 15 (blue lines) samples.

The beverage also shows high levels of total phenolic compounds, flavonoids and anthocyanins, but low content of tannins (Table 2). In general, no significant difference was observed in secondary metabolites content in both powders. The polyphenols retention capacity of beverage after being spray-dried with addition of MD was compared to the concentration of *Z. punctata* extract and chilto juice incorporated into the beverage. After spray-drying, 92% of the total phenolic compounds and 100% of flavonoids were recovered in the powdered beverages. These results demonstrate the stability of phenolic compounds/flavonoids to drying conditions. Anthocyanins and hydrophilic dyes are specifically compatible with water-based gel formulations, such as MD, and are used as coating molecules for polar matrices. In the present work conditions, 58% of the anthocyanin incorporated through chilto juice into the beverage was recovered after drying. These results showed that anthocyanins are less stable under drying conditions with a high temperature as reported in other studies on degradation of anthocyanin by temperature [70,71]. FTIR spectra of freeze-dried powders are shown in Figure 4. The spectra of both beverage powders were characterized by the presence of absorption bands at 3325–3395 cm^−1^, 2920 cm^−1^, 1740–1700 cm^−1^ and 1600–800 cm^−1^ region, attributed to the stretching, bending and deformation vibrations of polyphenolic compounds [11,46].

### 2.5. Antioxidant Capacity

In the present paper, several methodologies [1,2,7,10] were used to determine the antioxidant capacity of each component and of beverages obtained with them. The SC_50_ values were defined as the concentration of antioxidant needed to reduce 50% of the initial free radical. The ABTS cation radical scavenging capacity of chilto juice (SC_50_ = 6.5 ± 0.02 µg GAE/mL) and Zp extract (SC_50_ = 2.8 ± 0.02 µg GAE/mL) in comparison with the powdered beverages are shown in Table 3. The lemon honey 12 °Brix did not show ABTS radical cation scavenging activity. The SC_50_ values of beverages on ABTS cation radical did not show significative difference with the SC_50_ values of Zp extract alone, but was lower than SC_50_ values of chilto juice. These data evinced that the beverages that contain 26% juice and 9% extract were more active than chilto juice and Zp extract alone. The capacity of the beverages to scavenge ABTS radical was similar to a phenolic enriched ethanolic extract obtained from chilto pulp [37]. The spray-dried beverages were also able to scavenge hydroxyl radical and hydrogen peroxide. A different antioxidant capacity on hydroxyl radical and hydrogen peroxide scavenging was detected by varying the dextrose equivalent in the Maltodextrin (10 and 15 DE). The beverage with MD-DE 15 was more active in both assays. Experimental data evinced that the beverages also show inhibitory capacity on xanthine oxidase, the enzyme responsible for the production of uric acid and superoxide anion. The overproduction of such an enzyme produces an oxidative stress-associated inflammation process called gout with tissue damage by precipitation of urate. For these reasons, the XOD inhibitors act as antioxidants and anti-gouty. The beverage with MD-DE 10 was more active as the XOD inhibitor. The obtained results indicated that both formulations have antioxidant and anti-gout properties, but the one made with DE 15 has greater antioxidant power than DE 10, which could be due to its greater retention of sugars, citric acid and flavonoids.

### 2.6. Physicochemical and Flow Properties of Beverage Powder

The physicochemical properties of the powders are shown in Table 4. Some of these properties, highly dependent on the encapsulating material and the operative process conditions, provide predictions about the stability of the powders. The moisture content of spray-dried powder using 15% of maltodextrin was low and fails to show significant differences between DE 10 and 15. High water activity (a_w_) indicates more free water available for chemical reactions and growth of microorganisms, and hence shorter shelf life. The a_w_ for the obtained powders is within the normal range for atomized products, and within the limit to ensure powder stability (<0.3) [72]. However, the glass transition temperature (T_g_) was slightly above 25 °C and the powder hygroscopicity was high (26.69–26.33 g H_2_O/100 g DW), if the reported range for other spray-dried plant extracts or juices are considered [73]. Therefore, although microparticles have left the spray-dryer in a glassy state, the powder should be maintained below room temperature in adequate packaging conditions to avoid physical deterioration during handling and storage. The relatively low T_g_ values (near 30 °C) could be due to the sugar concentration generated in the beverage by the honey addition.

The powders exhibited high water solubility (97–98%). According to Chen and Patel (2008) [74], solubility is an important criterion to evaluate the product’s behavior in the aqueous phase since food powders require good solubility to be useful and functional. 

The colour parameters L* and a* failed to show significant differences (*p*-value < 0.05) between different DE of MDs, indicating the wall materials had a similar effect on the chromatic properties of the powder. Significant differences were observed in the b*, chroma and hue parameters. The beverage with DE 15 showed higher b* and hue values than the beverage with DE 10. These could explain the more orange tones to the beverage with DE 15, and more pinks to the beverage with DE 10. The high luminosity values can be mainly ascribed to MD, which is white in the dry state. The anthocyanin pigments of chilto juice provided the reddish tones to the formulation (Figure 1, Table 1).

Bulk and tap densities ranged from 0.37 to 0.38 g/cm^3^ and 0.505 and 0.508 g/cm^3^, respectively, conditions which are particularly desirable for the transport process and product packaging. The Hausner ratio and the Carr’s compressibility index values are suggestive of intermediate-flowing powder [54].

**Figure 5:**
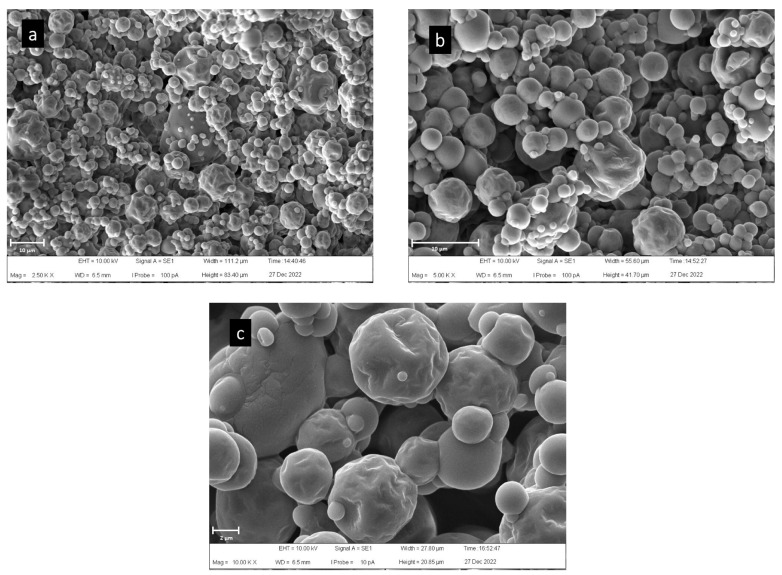
SEM micrographs of spray-dried beverage powder at 2500× (**a**), 5000× (**b**) and 10,000× (**c**) magnification.

Regarding particle size distribution studies, it was observed that powders exhibited a median (D_50_) between 10 and 11 μm, and a relatively broad size distribution (span values greater than 2), which is in accordance with the observations of the external morphology of the particles carried out by SEM (Figure 5). A heterogeneous distribution of spherical particles was obtained after spray-drying; small particles with a smooth surface and other bigger particles with some degree of shrinkage were present (Figure 5a,b), as a consequence of the rapid removal of water during the process. At higher magnifications (Figure 5c) a closer contact between particles is observed when compared with other previously reported spray-dried formulations containing fruit juice, such as blackberry, blackcurrant, raspberry and elderberry [75,76]. Although these formulations also included MD as the carrier, it was used at greater proportions (20–40%), thus preventing most particles from sticking. As was previously mentioned, a greater concentration of sugars from honey could have contributed to the decrease in the glass transition temperature (T_g_), thus causing particle agglomeration, affecting, to a certain extent, the powder physical properties and stability.

### 2.7. Toxicity

The Ames test has a highly predictive effect of carcinogenicity (around 80%) through diverse mechanisms, such as point mutations, base-pair substitutions (detected with TA100), or frameshift mutations (recognized with TA98). For this reason, the mutagenic effect of the extract, juice and beverages was evaluated to ensure its safe use. No mutagenic effects against the TA98 or TA100 strains up to 500 µg GAE/plate was observed, thus indicating the absence of direct mutagens in its composition (Table 5). Furthermore, the mutagenicity ratio (MR) values were lower than 2, thus indicating the absence of toxicity towards the genetic material of the strains. The same results were reported for Zp infusions, Zp ethanolic extracts and red chilto pulp [15,27,36,37,38]. Additionally, antigenotoxic activity was also reported for Zp extracts [15,27]. Zp infusions showed a cytotoxic effect against mammary, uterus and brain tumoral cells [77].

The acute toxicity of the beverages, using brine shrimp larvae as a model organism, was evaluated. No beverage was toxic up to concentrations of 500 µg GAE/mL.

## 3. Material and Methods

### 3.1. Juice Preparation

Juice from *S. betaceum* fruits was performed. Briefly, 1 kg of fully ripe fruits (orange red, “Sangre de buey”, Voucher number 617.907/LIL, Herbarium of ‘‘Fundación Miguel Lillo, 24°10′1.5″ S 65°23′40.3″ W) were processed to obtain 870 mL of juice. Only ripe undamaged fruits were employed. The fruits were manually peeled, and the pulp was homogenized using a blender coupled with a stainless-steel strainer (Philips Essence HR1357, Pekín, China) in order to remove the seeds. Then, the juice was used for the preparation of beverages. The juice was named red chilto juice.

### 3.2. Zuccagnia punctata Extract Preparation

The aerial parts of *Z. punctata* Cav (voucher specimen N° 605,935/LIL, Herbarium of ‘‘Fundación Miguel Lillo”) was collected from the Monte region of Argentina. The plant samples were dried at 40 °C, grounded, macerated and extracted with ethanol 5% (5, 10, 20 and 50% *w*/*v*) to obtain *Z. punctata* extract (Zp Extract). Zp extracts 20% (*w*/*v*) was selected to obtain beverage. Ethanol was purchased from Cicarelli, Santa Fe, Argentina.

### 3.3. Honey 12 °Brix Preparation

Lemon honey was obtained from local market in Tucumán, Argentina (Cooperativa Apícola Norte Grande, Tucumán, Argentina). Honey 12 °Brix was prepared by mixing with distilled water with 200 rpm stirring. The percentage of soluble solids was selected according to the requirements of the Argentine food code for non-alcoholic beverages [62].

### 3.4. Beverage Preparation

*S. betaceum* juice (26%), *Z. punctata* extract 20% (*w*/*v*) (9%), Maltodextrin DE 10 or 15 (15%) (Ingredion, Buenos Aires, Argentina), and honey (12 °Brix) (sweetener) were used to prepare 100 mL of beverage. The ingredients were mixed in a magnetic stirrer (Dlab MS-PA) for 10 min at 1000 rpm. The percentage of juice and extract was selected according to the requirements of the Argentine Food Code for non-alcoholic beverages [62].

### 3.5. Spray-Drying Process

The beverage was dried by using a spray-dryer (Buchi Modelo B-290, Flawil, Switzerland). The air flow type was concurrent, and inlet and outlet air temperatures were 130 °C and 65 °C, respectively. The liquid feed rate was 11 mL.min^−1^ and drying air flow (aspirator) rate was 600 L. h^−1^. The air flow in the drying chamber was 37 m^3^.h^−1^. The final product was vacuum sealed in polyethylene bags. The bags were then stored in a desiccator containing silica gel before quality evaluation.

### 3.6. Determination of Chemical Composition

#### 3.6.1. Total Polyphenol and Flavonoid Quantification

The total phenolic compound content of Zp extract 20% (*w*/*v*), red chilto juice, honey 12 °Brix and beverage was determined by using Folin–Ciocalteu reagent (Sigma Aldrich, St. Louis, MO, USA) [78]. Total flavonoids were estimated by using the Woisky and Salatino method (1998) [79]. Results were expressed as μg of gallic acid equivalent (GAE) per mL (μgGAE/mL) and quercetin equivalents (QE) per mL (μg QE/mL), respectively. Hydrolyzed and condensed tannins were quantified according to Carabajal et al., 2020 [10].

#### 3.6.2. Reducing and Total Sugar Quantification

Reducing sugars and total sugar of Zp extract 20% (*w*/*v*), red chilto juice, honey 12 °Brix and beverage were determined by using the Somogyi–Nelson method [80,81] and Phenol sulfuric method [82], respectively, for each powder. The experiment was performed in triplicate and the results were expressed as glucose equivalent (g GE/mL).

#### 3.6.3. HPLC-RID-DAD Analysis

A chemical profile of phenolic compounds of *Z. punctata* extract, red chilto juice and beverages was determined by HPLC. The dry extract (1 mg) was dissolved in 1 mL methanol (Biopack, Buenos Aires, Argentina) and filtered through a 0.45 mm nylon filter prior to injection of 20 mL. HPLC analysis was performed by using a Waters 1525 Binary Pumps system with a Waters 1500 Series Column Heater and a Waters 2998 photodiode array detector (PDA). A YMC—C18 column (250–4.6 mm, i.d. 5 mm) at 32 °C, a binary gradient solvent system consisting of solvent A (0.1% acetic acid (Biopack, Buenos Aires, Argentina) in water) and solvent B (methanol) starting with 10% B (0–35 min), 10 to 57% (35–45 min), 100% (45–65 min) and a flow rate of 1.0 mL/min were used for separation. PDA acquisitions were performed from 200 to 500 nm, and a chromatogram was integrated at 290 nm. The identification of individual compounds was made by comparison of retention times and UV spectral data with commercial standards (Sigma-Aldrich).

Sucrose, glucose and fructose content, as well as malic and citric acids were measured in the powders. The aqueous extracts were prepared by mixing 1 g of each powder with 12.5 mL of distilled water, with constant agitation for 5 min. Then, each sample was filtered and the remaining solid was again extracted by using the same procedure. Finally, more distilled water was added to reach a final volume of 25 mL. Soluble solids were determined, and samples were diluted to achieve 2° Brix. Before HPLC analysis, the aqueous extracts were filtered with a 0.2 µm Nylon filter (Genbiotech SRL, Buenos Aires, Argentina) into a vial.

Glucose, fructose and organic acids were separated by injecting 5 µL of extract in an Agilent 1260 HPLC (Agilent Techonologies, Santa Clara, CA, USA) equipped with an automatic injector (ALS) and two detectors: a diode array detector (DAD) for organic acid analysis and a refractive index detector (RID) for sugar analysis. Separation was performed by using a Hiplex H column (300 × 7.7 mm, 8 mm particle size, Agilent Technologies, Santa Clara, CA, USA) at 75 °C, and a mobile phase composed of 0.001 M H_2_SO_4_ with a flow rate of 0.4 mL/min (isocratic). For sucrose quantification, a ZORBAX carbohydrate column (150 × 4.66 mm, 5 µm particle size, Agilent Technologies, Santa Clara, CA, USA) and a mobile phase composed of 75% Acetonitrile and 25% Milli-Q water, were used. The analysis was performed at 25 °C with a flow rate of 1 mL/min. Chromatograms were recorded at 214 nm and calibration curves were created by using the aforementioned standards with a high linearity (r^2^ > 0.999).

### 3.7. Antioxidant Activity

#### 3.7.1. ABTS Radical Cation Decolorization Assay

The antioxidant capacity assay of Zp extract 20% (*w*/*v*), red chilto juice, honey 12 °Brix and beverages was carried out by the improved ABTS^•+^ (Sigma Aldrich, St. Louis, MO, USA) spectrophotometric method [83]. In this method, ABTS^•+^ solution was mixed with different quantities of chilto juice, Zp extract, honey 12 °Brix and beverage powder. Absorbance was recorded at 734 nm after 6 min. Results are expressed as SC_50_ values (μg/mL). SC_50_ (μgGAE/mL) was defined as the concentration of phenolic compounds necessary to scavenge 50% of ABTS free radicals. Quercetin was used as a reference compound.

#### 3.7.2. Hydrogen Peroxide (H_2_O_2_) Scavenging

The H_2_O_2_ (Cicarelli, Santa Fe, Argentina) scavenging was assessed according to Fernando and Soysa (2015) [84]. The reaction mixture contained phenol (Cicarelli, Origin in USA, packed in Santa Fe, Argentina) (12 mM), 4-aminoantipyrine 0.5 mM) (Sigma Aldrich, St. Louis, MO, USA), H_2_O_2_ (0.7 mM), sodium phosphate buffer (Cicarelli, Santa Fe, Argentina) (84 mM) at pH 7 and different concentrations of the chilto juice, Zp extract and beverage powder. It was kept at 35 °C for 20 min. Then, horseradish peroxidase (0.1 U/mL, Sigma Aldrich, St. Louis, MO, USA) was added and it was incubated at 37 °C for 30 min. The absorbance was measured at 504 nm. Results are expressed as SC_50_ values in μg GAE/mL. Quercetin (Sigma Aldrich, St. Louis, MO, USA) was used as a reference compound.

#### 3.7.3. 2-deoxy-D-ribose Degradation Assay

In order to evaluate the hydroxyl radical scavenging capacity of the beverage, the 2-deoxy-D-ribose degradation assay was applied as described by Chobot (2010) [85]. After the Fenton reaction, 250 μL of 2-thiobarbituric acid (1% *w*/*v*) (Sigma-Aldrich, Darmstadt, Germany) dissolved in trichloroacetic acid (3% *w*/*v*) (Cicarelli, Santa Fe, Argentina) was added to each vial to detect malondialdehyde (MDA). The tubes were vortexed and heated at 100 °C for 20 min. The reaction was halted by transferring the tubes into an ice water bath. Absorbance was determined at 532 nm. Results are expressed as SC_50_ values in μg GAE/mL.

#### 3.7.4. Xanthine Oxidase Assay

The effect of different aliquots of extracts and juice or beverage on the activity of xanthine oxidase (0.003 U) was determined spectrophotometrically at 290 nm (Microplate Reader Thermo Scientific Multiskan GO, Vantaa, Finland) by measuring the synthesis of uric acid from xanthine as substrate (60 μL, 1 mM). The reaction mixture (120 μL) was incubated at 25 °C for 30 min [86]. Indomethacin (20–100 µg/mL), acetylsalicylic acid (2–40 µg/mL) and allopurinol (2–100 µg/mL) were used as positive controls. Results are expressed as IC_50_ values in μg GAE/mL. IC_50_ (μg GAE/mL) was defined as the concentration of phenolic compounds necessary to inhibit 50% of enzyme activity. All reagents were purchased in Sigma Aldrich, St. Louis, MO, USA.

### 3.8. Physicochemical Properties

#### 3.8.1. Moisture, Soluble Solid Content and Water Activity

The beverage powder was characterized according to AOAC methods [81]: moisture, soluble solids and pH. Water activity was determined (a_w_) at 25 ± 1 °C by using an electronic dew-point water activity meter Aqualab Series 3 TE (Decagon Devices, Pullman, WA, USA), calibrated with saturated saline aqueous solutions.

#### 3.8.2. Glass Transition Temperature (Tg)

Glass transitions were determined by differential scanning calorimetry (DSC; onset values) by using a DSC 822e Mettler Toledo calorimeter (Schwerzenbach, Switzerland). The thermograms were evaluated by using the Mettler Stare program [87].

#### 3.8.3. Superficial Colour

Superficial colour was determined by measuring tristimulus parameters (CIELAB colour space) with a photocolorimeter model CR 400 (Minolta, Tokyo, Japan) using illuminant C and 2° observer angle. L* (brightness/darkness), a* (redness/greenness), and b* (yellowness/blueness) values were recorded. Measurements were performed using glass vials containing enough powder to complete 1 cm height. A white cylindrical cup was used to cover the vial and standardize the measurements.

These numerical values were converted into “chroma” (C*_ab_) and “hue angle” (h_ab_) parameters; the following equations were used:

C*_ab_ = (a*^2^ + b*^2^)^1/2^

h_ab_ = arctan (b*/a*)

#### 3.8.4. Solubility

Solubility was calculated according to Gagneten [73]. Powder samples (0.5 g) were dissolved in 50 mL of distilled water and then centrifuged (Bioamerican Science, Argentina) at 3000× *g* for 5 min. 10 mL of supernatant was transferred to a glass capsule and dried in an air-oven at 105 °C until constant weight. Solubility (%) was calculated by weight difference.

#### 3.8.5. Bulk and Compacted Density, Flowability (Carr Index) and Cohesiveness (Hausner Ratio)

Bulk and compacted density (g/mL) was determined according to Gallo et al., [88] with some modifications. The Hausner ratio (H) was related to the powder cohesiveness where levels below 1.2 are considered low, between 1.2–1.4, intermediate and above 1.5, high. Carr’s compressibility index (CI) was determined. The following scale was used: (a) CI = 10 excellent, (b) 11 < CI < 15 good; (c) 16 < CI < 20 fair; (d) 21 < CI < 25 acceptable; and (e) 26 < CI < 31 poor.

#### 3.8.6. Hygroscopicity

Hygroscopicity was determined in a closed desiccator at 20 °C containing a saturated solution of NaCl (Cicarelli, Santa Fe, Argentina) (75% RH). Duplicate samples were weighed periodically for twenty days, and hygroscopicity was expressed as the average of grams of adsorbed water per 100 g of dry matter (g aw/100 g dm).

### 3.9. Particle Morphology

Scanning electron microscopy (SEM) was applied to study microstructural characteristics of spray-dried particles by using a Zeiss microscope Supra 40 (Carl Zeiss, Oberkochen, Germany). Samples were placed on an aluminum support using conductive carbon doubled-sided adhesive tape, and coated by using a sputter coating machine model 108 (Cressington Scientific Instruments, Watford, UK) with gold nanoparticles. Micrographs were taken by using an acceleration voltage of 10.00 kV at a magnification range between 2500–10,000×.

Particle size distribution was measured according to Sette et al. 2022 [76], with a laser light diffraction instrument LA-950 V2 (Horiba, Kyoto, Japan) and using the dry powder method. The distribution width was characterized in terms of span index:

Span = (D_90_ − D_10_)/D_50_

where D_90_, D_50_, and D_10_ are the diameters for which the mass of the particle population is below 90%, 50%, and 10%, respectively. Values close to 1 of the span indicate a narrower particle distribution.

### 3.10. Toxicity

#### 3.10.1. Acute Toxicity

The toxicity test using *A. salina* has proven to be highly advantageous due to its simplicity and low cost, its good correlation with other animal testing methods, and the possibility of evaluating a large number of samples at the same time in a short period of time. Increasing concentrations of beverage (125–500 µg GAE/mL) were used to determine its acute toxic effect by utilizing the *Artemia salina* microplate assay [89]. Positive controls with potassium dichromate (Sigma-Aldrich, Darmstadt, Germany) (10–40 µg/mL) were assayed. After 24 h of exposition, the number of dead shrimps in each well was counted.

#### 3.10.2. *Salmonella typhymurium* Assay

***Toxicity Assay***. To examine the effects on the viability of *Salmonella Typhimurium* strains TA98 and TA100, a concentration range of extract (0.05–500 µg GAE/plate) was added to overnight-cultured *Salmonella typhimurium* strains TA98 or TA100 (0.1 mL) and S9 mix (0.5 mL) or 0.1 M phosphate buffer, pH 7 (0.5 mL), instead of S9 mix. The mixture was poured onto nutrient agar (Britania, Ciudad Autónoma de Buenos Aires, Argentina) plates. The plates were incubated at 37 °C for 2 days, and the number of colonies was counted. The beverages were then tested for their mutagenic potency exclusively in the nontoxic concentration range [90].

***Mutagenicity Assay***. *Salmonella Typhimurium* strains TA98 and TA100 were used to evaluate the possible mutagenic effect of the beverage (500 µGAE/plate) [84]. The 4-nitro-o-phenylenediamine (Sigma Aldrich, St. Louis, MO, USA) reagent (4-NPD, 5 µg/plate) was used as a positive control. All experiments were analyzed in triplicate with at least two replicates. The results were expressed as the number of revertants/plate, and the Mutagenicity Ratio (MR), that is, the ratio between the number of test plate revertants (induced revertant, IR) and the number of revertants on the control plate (spontaneous revertant, ER): MR = IR/ER was also calculated. The samples were considered mutagenic when the revertant average number was at least twice as much or higher than the spontaneous revertants, or if the MR was above two [90].

### 3.11. Statistical Analysis

For the statistical analysis of the data, the Tukey test was applied, with a level of significance *p* > 0.05, using the statistical package InfoStat V1.1 [91].

## 4. Conclusions

Non-conventional antioxidant beverages that include plant ingredients such as *Z. punctata* and *S. betaceum* as integral components are viable options within food science, whose aim is to provide benefits to human health. The production of these beverages requires, on one hand, the extraction of phenolics such as rosmarinic acid and derivates, chalcones and anthocyanins with demonstrated biological activity and, on the other, its combination to obtain an antioxidant formulation and protecting health-related compounds. From a technological perspective, state-of-the-art techniques were employed to profile the bioactive compounds incorporated into the beverage and modern drying techniques. The formulations produced microparticles of high water solubility with adequate features for handling, transport and storage. Therefore, this paper highlights the promising potential of botanical ingredients as an additional source of bioactive compounds to increased antioxidant activity in the special beverage segment.

## Figures and Tables

**Table 1:** Chemical characterization of chilto fresh juice, jarilla extract and honey.

	Red Chilto Juice	*Z. punctata* Extract	*Honey 12 °Brix*
**Total sugars (mg GE/mL)**	79.43 ± 3.50	5.82 ± 0.10	151.52 ± 3.28
**Reducing sugars (mg GE/mL)**	56.69 ± 2.06	5.88 ± 0.04	143.75 ± 1.45
**Total phenolic compounds** **(mgGAE/mL)**	1.02 ± 0.01	6.68 ± 0.22	0.03 ± 0.01
**Total Flavonoids** **(mg QE/mL)**	0.66 ± 0.02	12.10 ± 0.06	ND
**Condensed tannins** **(mg PB_2_E/mL)**	1.79 ± 0.02	38.14 ± 1.60	ND
**Hydrolysable tannins (ug/mL)**	4.67 ± 0.06	14.38 ± 0.05	ND
**Anthocyanins (mg C-3GE/mL** **)**	0.17 ± 0.01	ND	ND
2′, 4′-dihydroxy-3′-methoxy chalcone (µg/mL)	-	11.28 ± 0.04	-
2′, 4′-dihydroxychalcone (µg/mL)	-	23.22 ± 0.04	-
3-caffeoylquinic acid (µg/mL)	33.67 ± 0.03	-	-
Rosmarinic acid(µg/mL)	73.62 ± 0.02	-	-

GAE: Gallic acid equivalent; QE: quercetin equivalent; GE: glucose equivalent; PB2: Procyanidin B2 equivalent; C-3GE: Cyanidin 3 glucoside equivalent. ND: non detected. Values are reported as mean ± standard deviation of triplicates.

**Table 2:** Chemical characterization of antioxidant beverage powder.

	Spray-Dried Beverage	
	DE 15	DE 10
**Total sugars (g GE/g powder)**	1.81 ± 0.01 ^b^	1.08 ± 0.03 ^a^
**Reducing sugars (g GE/g powder)**	0.60 ± 0.03 ^a^	0.56 ± 0.02 ^a^
**Glucose mg/g powder**	38.00± 0.20 ^b^	30.00 ± 1.00 ^a^
**Fructose mg/g powder**	50.50 ± 0.80 ^b^	40.00 ± 2.00 ^a^
**Sucrose mg/g powder**	80.00 ± 3.00 ^b^	65.00 ± 2.00 ^a^
**Total proteins (mg ASB/g powder)**	2.02 ± 0.02 ^a^	2.26 ± 0.05 ^a^
**Citric acid (mg/g powder)**	5.40 ± 0.03 ^b^	3.10 ± 0.05 ^a^
**Malic acid (mg/g powder)**	1.10 ± 0.08 ^a^	0.90 ± 0.01 ^a^
**Total phenolic** **(mgGAE/g powder)**	2.92 ± 0.05 ^a^	2.89 ± 0.07 ^a^
**Flavonoids** **(mg QE/g powder)**	4.57 ± 0.19 ^b^	3.55 ± 0.14 ^a^
**Condensed tannins** **(mg PB_2_E/g powder)**	0.84 ± 0.08 ^a^	0.96 ± 0.06 ^a^
**Anthocyanins (mg C-3GE/g powder** **)**	0.07 ± 0.01 ^a^	0.09 ± 0.01 ^a^

DE 15: Beverage with Maltodextrin containing dextrose equivalent (DE) 15; DE 10: Beverage with Maltodextrin containing dextrose equivalent (DE) 10; GE: glucose equivalent; ASB: bovine serum albumin equivalent; GAE: gallic acid equivalent; QE: quercetin equivalent; GE: glucose equivalent; PB2E: procyanidin B2 equivalent; C-3GE: cyanidin 3-glucoside equivalent. Values are reported as mean ± standard deviation of triplicates. Different letters (a,b) in the same line indicate significant differences between formulation according to Tukey’s test (*p* ≤ 0.05).

**Table 3:** Antioxidant capacity of beverage containing chilto juice and Zp extract.

	Antioxidant Activity			
	ABTS^•+^	H_2_O_2_	HO•	XOD
**Spray-Dried Beverage**	**SC_50_ (µg GAE/mL)**	**SC_50_** **(µg GAE/mL)**	**SC_50_ (µg GAE/mL)**	**IC_50_ (µg GAE/mL)**
**DE 15**	3.29 ± 0.08 ^b^	17.00 ± 0.85 ^a^	32.82 ± 1.64 ^a^	114.43 ± 0.92 ^b^
**DE 10**	3.59 ± 0.02 ^b^	29.00 ± 1.45 ^b^	41.05 ± 2.05 ^b^	91.35 ± 0.86 ^a^

DE 15: Beverage with Maltodextrin containing dextrose equivalent (DE) 15; DE 10: Beverage with Maltodextrin containing dextrose equivalent (DE) 10; XOD: Xanthin oxidase; GAE: gallic acid equivalent. Values are reported as mean ± standard deviation of triplicates. SC_50_: Scavenging concentration 50% of free radicals; IC_50_: Inhibitory concentration 50% of XOD activity. Different letters (a,b) in the same column indicated significant differences between formulation according to Tukey’s test (*p* ≤ 0.05).

**Table 4:** Physicochemical characterization of spray-dried beverage of chilto and jarilla.

	Spray-Dried Beverage	
Properties	DE 15	DE 10
a_w_	0.248 ± 0.001 ^a^	0.227 ± 0.002 ^a^
Moisture (%)	6.030 ± 0.083 ^b^	5.070 ± 0.037 ^a^
pH	4.20 ^a^	4.15 ^a^
Total soluble solid (°Brix at 25 °C)	11 ^a^	11 ^a^
L*	82.65 ± 0.48 ^a^	82.74 ± 0.62 ^a^
a*	7.45 ± 0.28 ^a^	7.64 ± 0.22 ^a^
b*	12.19 ± 0.27 ^b^	8.91 ± 0.21 ^a^
Chroma	14.28 ± 0.36 ^b^	11.74 ± 0.29 ^a^
Hue angle	58.55 ± 0.53 ^b^	49.39 ± 0.34 ^a^
Bulk density (g/mL)	0.370 ± 0.005 ^a^	0.382 ± 0.008 ^a^
Compacted density (g/mL)	0.505 ± 0.015 ^a^	0.509 ± 0.010 ^a^
Hausner ratio	1.365 ± 0.055 ^a^	1.333 ± 0.010 ^a^
Carr Index	26.667 ± 0.15 ^a^	25.000 ± 0.015 ^a^
Tg (°C)	30.3 ± 1 ^a^	30.4 ± 1 ^a^
Hygroscopicity (g H_2_O/100 g DW)	26.69 ± 0.70 ^a^	26.33 ± 0.41 ^a^
Solubility	97.55 ± 0.49 ^b^	96.95 ± 0.21 ^a^
D_10_ (µm)	5.62 ± 0.20 ^a^	6.25 ± 0.45 ^b^
D_50_ (µm)	9.86 ± 0.26 ^a^	11.44 ± 0.94 ^b^
D_90_ (µm)	25.55 ± 2.95 ^a^	86.26 ± 6.75 ^b^
Span	2.02	6.99

DE 15: Beverage with Maltodextrin containing dextrose equivalent (DE) 15; DE 10: Beverage with Maltodextrin containing dextrose equivalent (DE) 10. Tg: transition temperature. L*: lightness, a* and b*: chromaticity. Values are reported as mean ± standard deviation of triplicates. Different letters (a,b) in the same line indicate significant differences between formulations according to Tukey’s test (*p* ≤ 0.05).

**Table 5:** Number of revertant colonies in the mutagenic Ames test.

Treatment	Vol/Concentration	n◦ Revertant TA98/Plate	n◦ Revertant TA100/Plate
Solvent control µL	100	38 ± 2 ^a^	111 ± 4 ^a^
4-nitro-o-phenylendiamine µg/plate	40	1488 ± 59 ^b^	960 ± 27 ^b^
*Zuccagnia punctata* extract µg/plate	125	32 ± 3 ^a^	115 ± 3 ^a^
	250	31 ± 3 ^a^	120 ± 3 ^a^
	500	33 ± 4 ^a^	111 ± 2 ^a^
Red chilto juice µg/plate	125	17 ± 2 ^a^	97 ± 5 ^a^
	250	19 ± 5 ^a^	102 ± 5 ^a^
	500	18 ± 2 ^a^	126 ± 5 ^a^
DE 15 µg/plate	125	28 ± 4 ^a^	120 ± 5 ^a^
	250	31 ± 4 ^a^	128 ± 3 ^a^
	500	41 ± 2 ^a^	124 ± 2 ^a^
DE 10 µg/plate	125	28 ± 4 ^a^	123 ± 4 ^a^
	250	31 ± 4 ^a^	131 ± 4 ^a^
	500	50 ± 3 ^a^	123 ± 3 ^a^

DE 15: Beverage with Maltodextrin containing dextrose equivalent (DE) 15; DE 10: Beverage with Maltodextrin containing dextrose equivalent (DE) 10. Means ± standard deviation followed by the same letter (a or b) in each column are not a significative difference (Tukey’s test *p* ≤ 0.05).

## Data Availability

Not applicable.

## Sample Availability

Samples are available from the authors.
